# Long-Term Antithyroid Drug Treatment of Graves’ Disease in Children and Adolescents: A 20-Year Single-Center Experience

**DOI:** 10.3389/fendo.2021.687834

**Published:** 2021-06-14

**Authors:** Ari Song, Su Jin Kim, Min-Sun Kim, Jiyeon Kim, Insung Kim, Ga Young Bae, Eunseop Seo, Young Seok Cho, Joon Young Choi, Sung Yoon Cho, Dong-Kyu Jin

**Affiliations:** ^1^ Department of Pediatrics, Incheon Sejong Hospital, Incheon, South Korea; ^2^ Department of Pediatrics, Inha University Hospital, Inha University College of Medicine, Incheon, South Korea; ^3^ Department of Pediatrics, Samsung Medical Center, Sungkyunkwan University School of Medicine, Seoul, South Korea; ^4^ Department of Nuclear Medicine and Molecular Imaging, Samsung Medical Center, Sungkyunkwan University School of Medicine, Seoul, South Korea

**Keywords:** Graves’ disease, hyperthyroidism, antithyroid drugs, remission, children

## Abstract

**Background/purpose:**

Graves’ disease (GD) is the most common cause of thyrotoxicosis in children and adolescents. There is some debate regarding the optimal treatment and predicting factors of remission or relapse in children and adolescents with GD. In this study, we report a retrospective study of 195 children and adolescents with GD treated at a single tertiary institution in Korea.

**Methods:**

This study included children and adolescents with GD diagnosed before 19 years of age from January of 2000 to October of 2020. The diagnosis of GD was based on clinical features, high thyroxine (FT4), suppressed thyroid-stimulating hormone, and a positive titer of thyrotropin receptor antibodies. Remission was defined as maintenance of euthyroid status for more than six months after discontinuing antithyroid drug (ATD).

**Results:**

A total of 195 patients with GD were included in this study. The mean age at diagnosis was 12.9 ± 3.2 years, and 162 patients (83.1%) were female. Among all 195 patients, five underwent thyroidectomy and three underwent radioactive iodine therapy. The mean duration of follow-up and ATD treatment were 5.9 ± 3.8 years and 4.7 ± 3.4 years, respectively. The cumulative remission rates were 3.3%, 19.6%, 34.1%, 43.5%, and 50.6% within 1, 3, 5, 7, and 10 years of starting ATD, respectively. FT4 level at diagnosis (P = 0.001) was predicting factors for remission [HR, 0.717 (95% CI, 0.591 – 0.870), P = 0.001]. Methimazole (MMI)-related adverse events (AEs) occurred in 11.3% of patients, the most common of which were rash and hematologic abnormalities. Of a total of 26 AEs, 19 (73.1%) occurred within the first month of taking MMI.

**Conclusions:**

In this study, the cumulative remission rate increased according to the ATD treatment duration. Long-term MMI treatment is a useful treatment option before definite treatment in children and adolescents with GD.

## Introduction

Graves’ disease (GD) is an autoimmune disorder characterized by hyperthyroidism, diffuse goiter, and/or ophthalmopathy that is caused by the activation of the thyroid-stimulating hormone (TSH) receptor by thyrotropin receptor antibodies (TRAb). GD is the most common cause of thyrotoxicosis in children and adolescents and contributes to 10-15% of all pediatric thyroid disease (1). The incidence of GD in children and adolescents varies by country, ranging from 1 to 14 per 100,000 person-years. It is relatively rare in children compared to adults, as less than 5% of all GD patients are children (2). However, the incidence of GD in children and adolescents is currently estimated to be rising (3–5).

Treatment options of GD in children and adolescents include antithyroid drug (ATD), radioactive iodine (RAI) therapy, and thyroidectomy, which are the same as in adults. There is no consensus regarding the optimal treatment of GD in children and adolescents, and the subject remains a matter of debate (6–8). Definite treatments such as thyroidectomy and RAI therapy may cause permanent hypothyroidism, requiring life-long thyroid hormone replacement, and RAI therapy has the potential for oncogenic damage. For these reasons, the majority of clinicians choose ATD as the first-line treatment option (8–10).

Long-term ATD treatment may lead to the development of drug-related adverse effects (AEs), a high rate of relapse after drug discontinuation, and compliance problems. In children and adolescents, previous studies have focused on predicting factors of remission or relapse after ATD discontinuation (11–14). However, no congruous predicting factors have been determined yet. The treatment policy of GD in children and adolescents varies between countries and depends on local traditions and resources, patient age, the preferences of the patients and their parents, and disease severity. This is a retrospective study of 195 children and adolescents with GD treated at a single tertiary institution in Korea.

## Materials and Methods

### Patient Population and Data Collection

We reviewed the medical records of 195 patients diagnosed with GD before the age of 19 at Samsung Medical Center from January of 2000 to October of 2020. The diagnosis of GD was based on the presence of typical clinical features (tachycardia, tremor, exophthalmos, goiter, etc.), high levels of free thyroxine (FT4), suppressed levels of TSH, and a positive titer of TRAb. Exclusion criteria were as follows: (1) patients who had been followed up for less than six months, (2) patients treated with ATD for more than six months at another hospital, and (3) patients who had other thyroid diseases (e.g., chronic lymphocytic thyroiditis, papillary carcinoma, etc.). Clinical data such as age, sex, height, weight, body mass index (BMI), GD symptoms and signs, drug-related AEs, a personal history of non-thyroid autoimmune diseases, and family history of thyroid autoimmune diseases in first-degree relatives were collected retrospectively.

Goiter was evaluated as present or absent at physical examination. The absence of goiter was defined as Grade 0, and the presence of goiter was defined as Grade 1 or 2 according to the WHO goiter classification. Graves’ ophthalmopathy (GO) was evaluated as present or absent. Presence of GO was defined as abnormal ocular signs and symptoms (e.g., eyelid retraction, eyelid lag, exophthalmos) by an endocrinologist and an ophthalmologist if needed. Laboratory data included FT4, total triiodothyronine (T3), TSH, anti-thyroglobulin antibody (ATA), anti-microsomal antibody (AMA), TRAb, complete blood count (CBC), creatine kinase (CK), and liver function test (LFT). Two different TRAb assays were used over the study period. During the first part of the study (before March of 2011), we used a TRAK-Assay (Brahms, Berlin, Germany; reference range, < 10% negative, 10–15% borderline, > 15% positive), which is a first-generation assay and a conventional radioreceptor assay using porcine thyrocytes membrane. Thereafter (after March of 2011), we used a TRAK human RIA (Brahms; reference range, < 1.0 IU/L negative, 1.0–1.5 IU/L borderline, > 1.5 IU/L positive), which is a second-generation assay and a coated tube radioimmunoassay using recombinant human TSH receptor. Thyroid function test (TFT) was measured by radioimmunoassay using an RIA kit (Immunotech Inc., Praha, Czech Republic) or chemiluminescence immunoassay using ADVIA CENTAUR XP (Seimens Healthcare Diagnostics, Massachusetts, USA). For accurate statistical analysis, the TFT values were standardized considering the reference ranges of each analysis kit (supplementary data). Laboratory data, including TFT, CBC, and LFT, were regularly evaluated at least every 3 months during the follow-up period. This study was approved by the Institutional Review Board of Samsung Medical Center (SMC 2021-01-051).

### Treatment Protocols

All patients were initially treated with an ATD, including methimazole (MMI) or propylthiouracil (PTU), although MMI was primarily used in this study. The initial doses of MMI were 0.5 to 1.0 mg/kg/day, (maximum 30 mg/day), which were gradually reduced in a titration regimen and discontinued when euthyroidism was maintained at the lowest dose (2.5 mg/day) for 6-12 months. In this study, two pediatric endocrinologists made a decision according to the treatment policy of the center. Remission was defined as maintenance of euthyroid status for more than six months after discontinuation of ATD. Relapse was defined as elevated serum FT4 with suppressed TSH during the follow-up period after ATD withdrawal.

### Statistical Analysis

Continuous variables are presented as mean ± standard deviation (SD). Categorical variables are expressed as rates and proportions. Kaplan–Meier survival analysis was used to estimate the time to remission. A comparison of categorical variables was performed using the Chi-square test or the Fisher exact test (in case of expected frequencies < 5). A comparison of continuous variables was performed using the Mann-Whitney U test (nonparametric test). The Cox regression model was used to identify the significant predictors of remission. Total T3 was not included in the Cox regression model because of collinearity with free T4. The results are expressed as hazard ratios (HR) with 95% confidence intervals (CI). A P-value < 0.05 indicates statistical significance. Statistical analyses were performed using SPSS Statistics software, version 23 (IBM Corp., Armonk, NY, USA) and SAS version 9.4 (SAS Institute, Cary, NC, USA).

## Results

### Clinical Characteristics of Patients With GD

**Figure 1:**
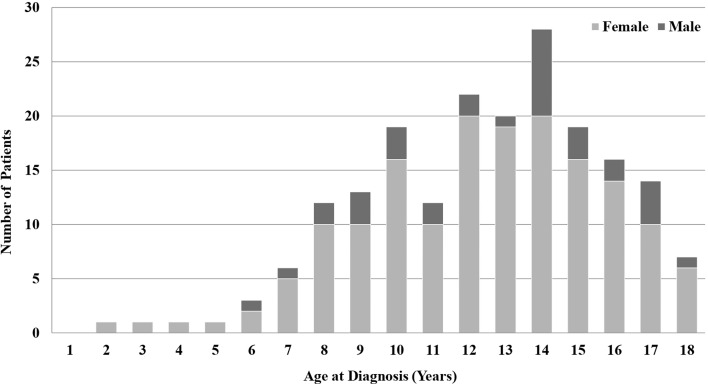
Distribution of patients with Graves’ disease according to sex and age at diagnosis.

Among the 195 patients, there were 162 (83.1%) females and 33 (16.9%) males. Their mean age at diagnosis was 12.9 ± 3.2 years (**Figure 1**). The mean duration of follow-up and ATD treatment were 5.9 ± 3.8 years and 4.7 ± 3.4 years, respectively. Among the 195 patients, 152 (77.9%) had a goiter and 64 (32.8%) had ophthalmopathy. Five patients (2.6%) had non-thyroid autoimmune diseases such as insulin-dependent diabetes mellitus or myasthenia gravis and one (0.5%) presented with thyrotoxic periodic paralysis (TPP). Among all subjects, 68 (34.9%) had a familial history of thyroid autoimmune diseases in first-degree relatives (**Table 1**).

**Table 1:** Clinical characteristics of patients with Graves’ disease.

Characteristics	All patients (*n* = 195)
Male/Female (%)	33 (16.9%)/162 (83.1%)
Age at diagnosis (years)	12.9 ± 3.2
BMI score at diagnosis	18.9 ± 3.4
Duration of follow-up (years)	5.9 ± 3.8
Duration of ATD treatment (years)	4.7 ± 3.4
Presence of goiter (%)	152 (77.9%)
Presence of ophthalmopathy (%)	64 (32.8%)
Past medical history of autoimmune disease (%)	5 (2.6%)
Familial history of autoimmune thyroid disease (%)	68 (34.9%)
Thyroid storm (%)	12 (6.2%)
Thyroidectomy (%)	5 (2.6%)
RAI (%)	3 (1.5%)

Data are expressed as mean ± SD.

BMI, body mass index; ATD, Antithyroid drugs; RAI, radioactive iodine.

### The Clinical Course of Patients With GD

**Figure 2:**
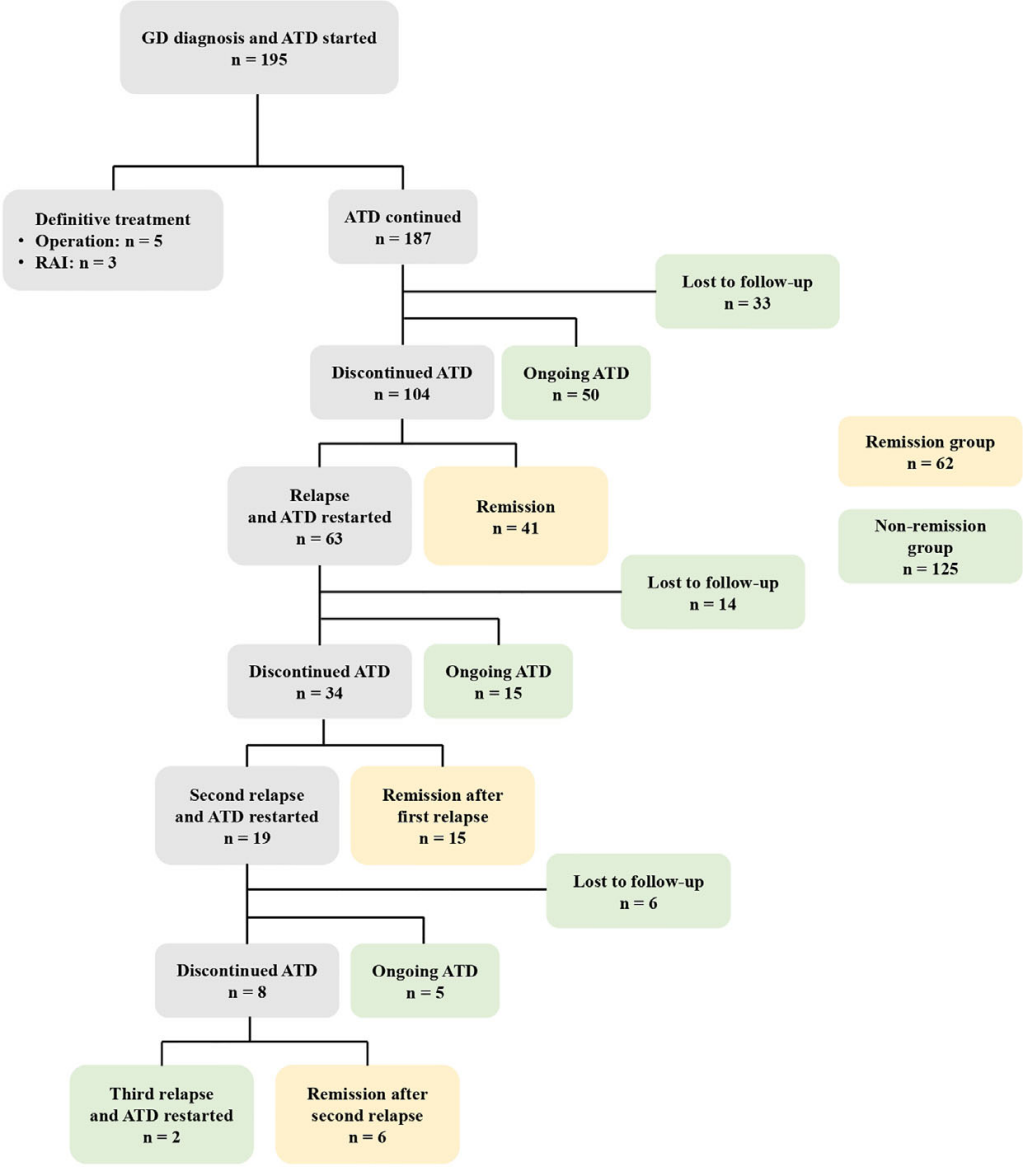
The clinical course of 195 patients with GD initially treated with ATD. GD, Graves’ disease; ATD, Antithyroid drugs; RAI, Radioactive iodine.

**Figure 2** demonstrates the clinical course of all patients. Among the 195 patients, eight (4.1%) underwent definite treatment: five thyroidectomies and three RAI therapies. Definite treatment was performed due to poorly controlled hyperthyroidism despite ATD treatment or was based on the patient’s preferences. Of the 187 patients treated with ATD, 50 (26.7%) have continued ATD treatment without remission, 104 (55.6%) discontinued ATD, and 33 (16.9%) were lost to follow-up. Of the 104 patients who discontinued ATD, 41 (39.4%) achieved remission while 63 (60.6%) relapsed. Of the 63 patients who experienced their first relapse, 15 (23.8%) continued ATD, 34 (54.0%) discontinued ATD, and 14 (22.2%) were lost to follow-up. Of the 34 patients who discontinued ATD, 19 patients experienced a second relapse and two experienced a third relapse.

Among the 187 patients treated with ATD, 41 achieved remission without relapse, 15 achieved remission after the first relapse, and six achieved remission after the second relapse. These 62 patients were defined as the remission group. The non-remission group (n = 125) included the 53 patients who were lost to follow-up, the 50 patients who continued ATD treatment without remission, the 15 patients who continued ATD after their first relapse, the five patients who continued ATD after their second relapse, and the two patients who continued ATD after their third relapse.

**Figure 3:**
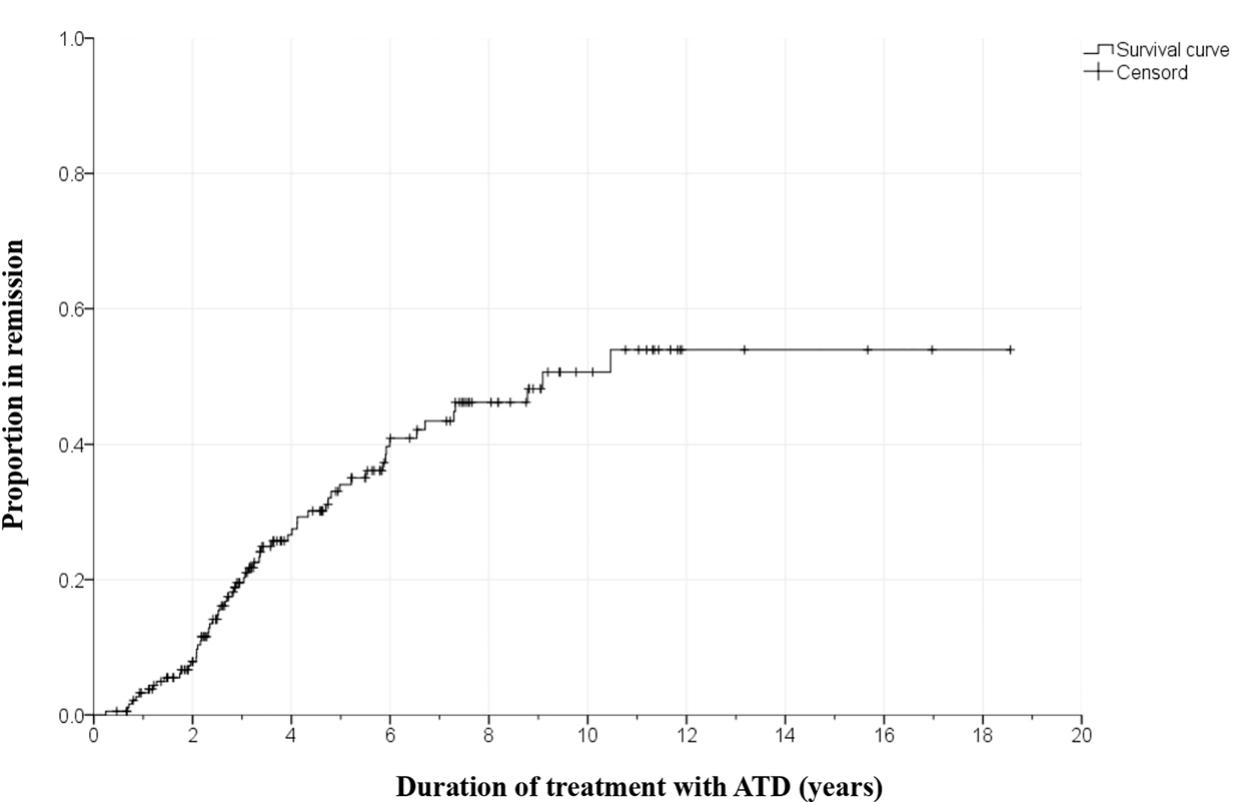
Kaplan-Meier survival curve showing time to remission (*n* = 187). ATD, Antithyroid drugs.

There were no significant differences in clinical and biochemical characteristics between the patients who were lost to follow-up and all patients (**Supplementary Table 1**). In the remission group, the time to achieve remission was 43.2 ± 26.4 months, and the time to relapse was 9.7 ± 13.4 months. In the 187 patients treated with ATD, Kaplan-Meier survival analyses predicted that the cumulative remission rates were 3.3%, 19.6%, 34.1%, 43.5%, and 50.6% within 1 year, 3 years, 5 years, 7 years, and 10 years, respectively (**Figure 3**).

### Comparison of the Remission Group *vs*. the Non-Remission Group

The total T3 level at diagnosis was higher in the non-remission group than in the remission group (502.6 ± 218.8 *vs.* 408.2 ± 192.7 pg/mL, P = 0.009). FT4 level at diagnosis was higher in the non-remission group than in the remission group (5.0 ± 1.9 *vs.* 3.9 ± 1.5 ng/dL, P = 0.002). Other variables, including sex, past medical history of autoimmune disease, family history of thyroid disease, ophthalmopathy, goiter, age at diagnosis, BMI score at diagnosis, ATA at diagnosis, and AMA at diagnosis, were not significantly different between the two groups (**Table 2**).

**Table 2:** Comparison of clinical and biochemical variables between the two groups.

Parameter	All patients (*n* = 187)	Remission (*n* = 62)	Non-Remission (*n* = 125)	*P*-value
Sex				0.566^=^
Male, *n* (%)	32 (17.1%)	12 (19.4%)	20 (16.0%)	
Female, *n* (%)	155 (82.9%)	50 (80.6%)	105 (84.0%)	
Past medical history of AID				0.334^*^
Positive, *n* (%)	5 (2.7%)	3 (4.8%)	2 (1.6%)	
Negative, *n* (%)	182 (97.3%)	59 (95.2%)	123 (98.4%)	
Family history of AITD				0.636^=^
Positive, *n* (%)	65 (34.8%)	23 (37.1%)	42 (33.6%)	
Negative, *n* (%)	122 (65.2%)	39 (62.9%)	83 (66.4%)	
Ophthalmopathy at diagnosis				0.766^=^
Yes, *n* (%)	60 (32.1%)	19 (30.6%)	41 (32.8%)	
No, *n* (%)	127 (67.9%)	43 (69.4%)	84 (67.2%)	
Goiter at diagnosis				0.144^=^
Yes, *n* (%)	145 (77.5%)	52 (83.9%)	93 (74.4%)	
No, *n* (%)	42 (22.5%)	10 (16.1%)	32 (25.6%)	
Age at diagnosis (years)	12.9 ± 3.2	12.7 ± 3.5	13.0 ± 3.0	0.585^+^
BMI score at diagnosis	18.9 ± 3.4	18.4 ± 3.8	19.1 ± 3.2	0.118^+^
Total T3 at diagnosis (pg/mL) (*n* = 147)	468.6 ± 214.0	408.2 ± 192.7	502.6 ± 218.8	0.009^+^
FT4 at diagnosis (ng/dL) (*n* = 137)	4.6 ± 1.9	3.9 ± 1.5	5.0 ± 1.9	0.002^+^
ATA at diagnosis (*n* = 146)				0.194^=^
Positive, *n* (%)	97 (66.4%)	31 (59.6%)	66 (70.2%)	
Negative, *n* (%)	49 (33.6%)	21 (40.4%)	28 (29.8%)	
AMA at diagnosis (*n* = 149)				0.261^=^
Positive, *n* (%)	112 (75.2%)	37 (69.8%)	75 (78.1%)	
Negative, *n* (%)	37 (24.8%)	16 (30.2%)	21 (21.9%)	

Data are expressed as mean ± SD.

^=^Chi-square test; ^*^Fisher’s exact test; ^+^Mann–Whitney U test.

AID, autoimmune disease; AITD, autoimmune thyroid disease; BMI, body mass index; T3, triiodothyronine; FT4, free thyroxine; ATA, anti-thyroglobulin antibody; AMA, anti-microsomal antibody.

### Predictors Associated With GD Remission

In the Cox regression model, the FT4 level at diagnosis [HR, 0.717 (95% CI, 0.591–0.870), P = 0.001] was identified as a predictive factor for GD remission in children and adolescents in this study (**Table 3**).

**Table 3:** Predicting factors associated with GD remission in children and adolescents.

Parameter	HR (95% CI)	*P*-value
Sex		
Male, *n* (%)	1.018 (0.542 – 1.914)	0.956
Female, *n* (%)		
Family history of AITD		
Positive, *n* (%)	1.054 (0.629 – 1.765)	0.843
Negative, *n* (%)		
Ophthalmopathy at diagnosis		
Yes, *n* (%)	0.827 (0.482 – 1.421)	0.492
No, *n* (%)		
Goiter at diagnosis		
Yes, *n* (%)	1.408 (0.716 – 2.772)	0.322
No, *n* (%)		
Age at diagnosis (years)	0.996 (0.918 – 1.081)	0.931
BMI score at diagnosis	0.952 (0.879 – 1.032)	0.232
FT4 at diagnosis (ng/dL) (*n* = 137)	0.717 (0.591 – 0.870)	0.001
ATA at diagnosis (*n* = 146)		
Positive, *n* (%)	0.862 (0.494 – 1.504)	0.601
Negative, *n* (%)		
AMA at diagnosis (*n* = 149)		
Positive, *n* (%)	0.654 (0.363 – 1.179)	0.158
Negative, *n* (%)		

Data are expressed as mean ± SD.

Cox regression model was used.

HR, hazard ratio; CI, confidence interval; AITD, autoimmune thyroid disease; BMI, body mass index; FT4, free thyroxine; ATA, anti-thyroglobulin antibody; AMA, anti-microsomal antibody.

### Drug-Related AEs

**Figure 4:**
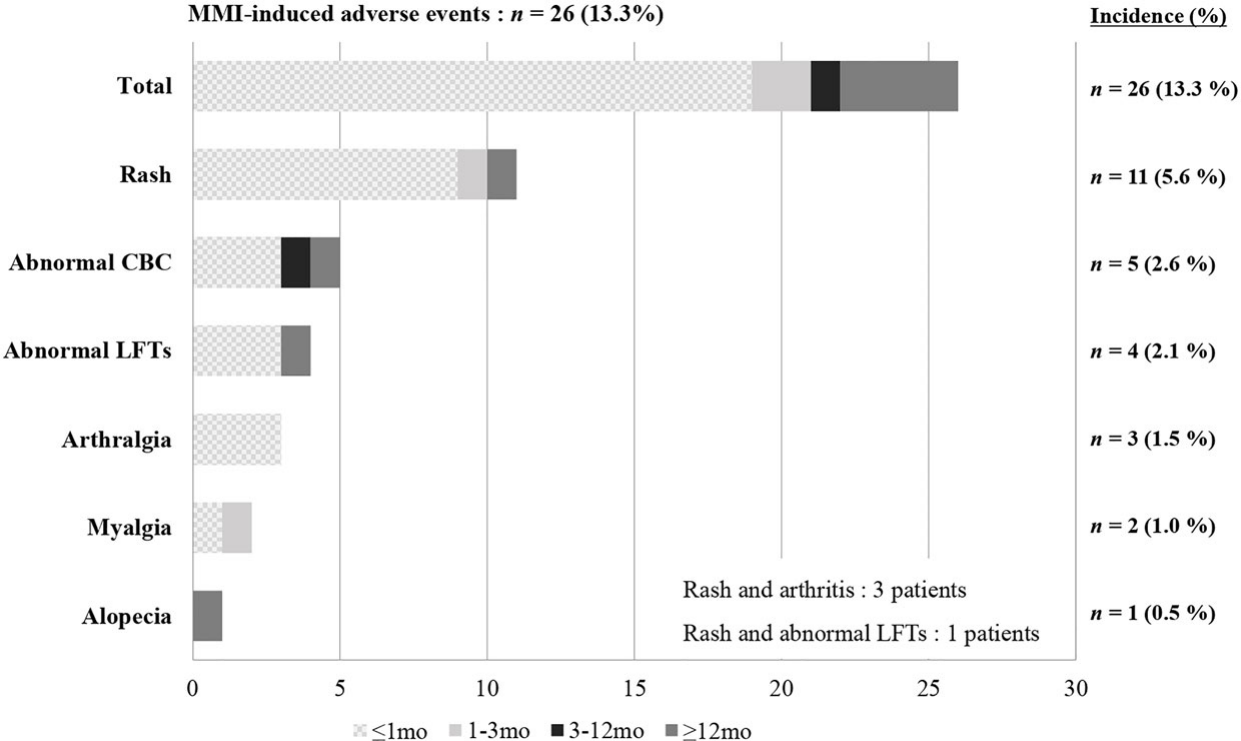
Incidence and time to onset of MMI-induced adverse events. MMI, Methimazole; CBC, Complete blood count; LFTs, Liver function tests.

Most patients in our study were initially treated with MMI, although 13 patients started PTU at another hospital and then changed to MMI at our center. Among the 195 patients who had taken MMI at least once, 22 (11.3%) experienced a total of 26 AEs (**Figure 4**). Rash was the most common AE reported (n = 11), followed by hematologic abnormalities (n = 5), elevated liver enzymes (n = 4), arthralgia (n = 3), myalgia with elevated CK (n = 2), and alopecia (n = 1). Among the 26 AEs, 19 occurred within one month of taking MMI and 22 occurred within the first three months. Five were late AEs that occurred after more than 3 months of taking MMI. These were rash, alopecia, abnormal LFTs, and two events of neutropenia, which occurred at 30 months, 3 years, 5 years, 11 months, and 6 years after taking MMI, respectively.

Rash occurred in most of the patients (9 of 11) within one month, and three patients with rash also reported arthralgia. The rash and arthralgia were suitably controlled by antihistamine drugs and non-steroidal anti-inflammatory drugs and spontaneously disappeared. However, one patient switched to PTU because both symptoms persisted despite medical therapy. Of the five patients with hematologic abnormalities, one patient showed severe agranulocytosis (absolute neutrophil count (ANC) to 50/μL) with fever and abdominal pain on the fifth day of taking MMI. They recovered immediately after stopping MMI. Also, four patients had a slightly decreased WBC count and neutropenia (ANC > 500/μL). LFT abnormalities and elevated CK were mild and recovered spontaneously.

## Discussion

This is a retrospective study of long-term ATD treatment in children and adolescents with GD at a single tertiary institution in Korea.

Generally, the remission rates for GD in children and adolescents are 21-49% (12, 15–17). These rates may vary according to the ATD regimen or definition of the term remission. The remission rate tends to increase as the duration of ATD administration increases, reaching up to 50% in several studies of more than 10 years. However, it does not appear to increase beyond that, and some research indicates that these rates plateau (15, 16, 18). In this study, the cumulative remission rate was 3.3% at 1 year after starting ATD treatment, 19.6% at 3 years, 34.1% at 5 years, 43.5% at 7 years, and 50.6% at 10 years. In the Kaplan-Meier survival curve, remission rates hardly increased after 10 years. This may be due to the small number of patients who were followed for more than 10 years.

Several previous studies on predicting factors associated with remission or relapse in children and adolescents with GD of a similar size or larger than this study are summarized in **Table 4**. The literature reports different predictors for GD remission or relapse in children and adolescents, such as high TRAb levels, high FT4 levels, goiter, age or BMI-SDS at time of diagnosis, and ethnicity. Kaguelidou et al. reported that high serum TRAb levels at diagnosis were associated with a higher risk of relapse (HR = 1.21 by 10 U, P = 0.03) in a prospective, multi-center cohort study of 154 children with GD (19). Gasaldi et al. reported that lower median TRAb levels at diagnosis (less than 2.5 times the upper limit of the reference range) might be associated with positive outcomes (P = 0.031) in a retrospective, multi-center study of 115 children with GD (12). In our study, the TRAb measurement method was changed during the study period, therefore, the analysis of each measurement method was performed separately. However, we could not find consistent significant differences in TRAb levels within each group (**Supplementary Table 2**).

**Table 4:** Literature review of predicting factors associated with GD remission or relapse in children and adolescents.

Author, year (reference)	Country	Study design	Number of patients (F/M)	Definition of Remission	Age at diagnosis (years)	Follow-up duration (years)	ATD treatment duration (years)	Remission rate	Predictors of remission	Odds ratio (95% CI), *P*-value
**This study**	Korea	Retrospective, single center	195 (162/33)	Euthyoroidism without ATD > 6 months	12.9 ± 3.2	5.9 ± 3.8	4.7 ± 3.4	3.3% (1 yr) 19.6% (3 yr) 34.1% (5 yr) 43.5% (7 yr) 50.6% (10 yr)	Goiter	3.53 (1.15-10.79) *P* = 0.027
									FT4 at diagnosis	0.63 (0.47-0.83) *P* = 0.001
**Glaser,** **1997 (** 17 **)**	USA	Retrospective, multi-center	191 (153/38)	Euthyoroidism without ATD > 6 months	12.1 ± 3.7	NA	2.4 ± 1.4	6% (1 yr) 25% (2 yr)	BMI-SDS	4.15 (1.63-10.59) *P* = 0.003
									Goiter size	0.22 (0.05-0.96) *P* = 0.043
**Kaguelidou,** **2008 (** 19 **)**	France	Prospective, multi-center	154 (118/36)	Relapse, hyperthyroidism without ATD	11.9 (9.4-13.9)	NA	2.0 ± 0.2	Relapse rate 59% (1 yr) 68% (2 yr)	Age	HR = 0.74 per 5 yr *P* = 0.03
									Non-Caucasian	HR = 2.54 *P* < 0.004
									High TRAb at diagnosis	HR = 1.21 by 10 U *P* = 0.003
									Duration of 1^st^ course of ATD	HR = 0.57 per 12m *P* = 0.005
**Leger,** **2012 (** 15 **)**	France	Prospective multi-center	154 (118/36)	Euthyoroidism without ATD > 18 months	11.9 (9.4-13.9)	10.4 (9.0-12.1)	NA	20% (4 yr) 37% (6 yr) 45% (8 yr) 49% (10 yr)	High FT4 at diagnosis	SHR = 0.4(0.2-0.8) *P* = 0.001
									Other AID	SHR = 2.2(1.2-4.2) *P* = 0.01
**Ohye,** **2014 (** 16 **)**	Japan	Retrospective, single center	1138 (995/143)	Euthyoroidism without ATD > 12 months	16 (range, 3-18)	NA	3.8 (range, 0.3-24.8)	9.3%,24.6%,34.6%, 38.9%,41.9%,45.2%, 46.2% (2,4,6,8,10,14,20 yr, respectively)	No significant predictor	
**Gastaldi,** **2014 (** 12 **)**	Italy	Retrospective, multi-center	115 (98/17)	Euthyroidism after 2yr of ATD	11.3 ± 3.5	6.5 ± 3.2	2.9 (2.0-4.0)	33% (2 yr)	Lower TRAb at diagnosis	*P* = 0.031
									Time for TRAb normalization	*P* = 0.026
**Rabon,** **2016 (** 18 **)**	USA	Retrospective, single center	291 (229/62)	Euthyoroidism without ATD > 3 months	12.3 ± 3.8	NA	NA	21% Plateus after 5.5 yr	No significant predictor	

Data are expressed as Mean ± SD or Median (interquartile range).

GD, Graves’ disease; F, female; M, male; ATD, Antithyroid drugs; CI, confidence interval; FT4, free thyroxine; NA, not available; BMI-SDS, body mass index-standard deviation score; HR, hazard ratio; AID, auto-immune disorder; SHR, sub-hazard ratio; TRAb, TSH-receptor antibody.

We found that a low FT4 level at diagnosis was a predictor of remission, which was consistent with some previous research. Leger et al. reported that less severe parameters at the time of diagnosis (patients with FT4 <35 pmol/liter) and the presence of other autoimmune diseases had an independent positive effect on remission rate (15).

However, Ohye reported that no significant predictors of remission were found in a large, single-center cohort study involving 1,138 children with GD that has been conducted to date (16). Another single-center study of 291 children with GD by Rabon et al. found no significant factors predicting remission (18).

There is a debate about the appropriate treatment policy for GD in children and adolescents (6, 7, 20, 21). In a Cochrane review involving 3,388 adults with GD in 26 randomized trials published in 2010, the optimal duration of ATD treatment was 12-18 months (22). However, recently published studies suggest long-term medical therapy with the lowest dose of ATD is effective and safe in both adults and children (21, 23–27). A recent review article in children with GD suggested that ATD treatment maintains normal homeostasis of the hypothalamus-pituitary-thyroid axis and that long-term ATD treatment with the lowest dose of MMI/carbimazole (CMZ) should be offered to increase the possibility of remission (21). Azizi et al. reported that long-term MMI treatment for a mean of 9.1 years is a safer and more effective treatment option than short-term MMI treatment in a randomized, parallel-group trial of 66 adolescent patients with GD (24). In an ongoing study, 59 patients underwent very long-term ATD treatment with low-dose MMI for an average of 14.2 years, and most had euthyroidism without significant MMI-related AEs (25). There are few studies with a mean duration of ATD treatment of four years or more in children and adolescents with GD (15, 24, 28, 29). In our study, patients received relatively long-term MMI treatment for a mean of 4.7 ± 3.4 years and the remission rate increased according to the duration of MMI treatment in the Kaplan-Meier graph.

In long-term ATD treatment, the appropriate dosage, administration duration, and AEs of drugs are important issues. The American Thyroid Association and the American Association of Clinical Endocrinologists recommend that MMI be used to treat GD in children because PTU sometimes causes severe AEs, such as liver failure and antineutrophil cytoplasmic antibody (ANCA)-associated vasculitis (30). In our study, MMI was mainly used for ATD treatment, except in cases of intolerable MMI-related AEs. The American Thyroid Association guidelines published in 2016 recommend that the initial MMI dose be 0.2-0.5 mg/kg, with a range of 0.1-1.0 mg/kg/day (30). However, treatment strategies vary between researchers and countries. In Japan, most studies used an initial dose of 1mg/kg/day (16, 31). Kaguelidou et al. used 0.5-0.7mg/kg/day (19), and Rabon et al. used 0.2-0.8 mg/kg/day (18). Several studies have reported that the AEs of MMI occur more frequently with higher doses than lower doses, and most occur early in treatment (29, 32, 33). The AEs of MMI include minor allergic reactions such as rash and serious AEs such as agranulocytosis, vasculitis, and hepatic damage. In adults with GD, reported AEs of MMI were cutaneous allergic reaction (13-24%), increased liver enzymes (2.7-3.8%), and agranulocytosis (0.3-0.7%) (33–36). In children and adolescents with GD, the prevalence of MMI-related AEs varied (6-35%), but fatal AEs were rare, and most occurred within the first three months (16, 19, 20, 37). Accurate prevalence data for agranulocytosis in children and adolescents taking MMI have not yet been reported (30).

In our study, MMI was initially dosed at 0.5-1.0 mg/kg/day, depending on the patient’s FT4 level at diagnosis, and was maintained at the lowest dose of 2.5 mg/day after euthyroidism. MMI-related AEs occurred in 11.3% of all patients, the most common of which were rash and hematologic abnormalities, followed by increased liver enzymes and arthralgia. The rash and arthralgia were suitably controlled by medical therapy, and the increased liver enzymes were within five times the normal range, with no associated jaundice. Most AEs disappeared spontaneously. Serious agranulocytosis occurred on the fifth day of MMI treatment in one patient (0.5% of all patients) who recovered immediately after drug discontinuation. The majority of AEs (19 cases, 73.0%) occurred within the first month, and only four cases (15.4%) occurred after one year of treatment. Therefore, careful monitoring to detect AEs is imperative, especially early in MMI treatment. It is essential to use the lowest MMI dose to increase the chance of remission and minimize side effects.

This study has several limitations. One is that 28.3% of patients were lost to follow-up over the 20-year study period. Some of these patients likely became adults and were transferred from pediatric endocrinologists to endocrinologists. However, those lost to follow-up showed similar baseline clinical and biochemical characteristics compared with those who remained in the study (**Supplementary Table 1**). Another limitation is the insufficient number of patients to identify predicting factors of remission. TRAb was not included in the statistical results due to a change in the measurement method during the study. Nevertheless, the main strength of this study is that the safety of long-term MMI treatment was assessed with a mean treatment duration of 4.7 ± 3.4 years and was conducted with the same regimen for 20 years at a single institution.

## Conclusion

In this study, the cumulative remission rate increased according to the ATD treatment duration. The drug-related AEs usually occur early in treatment, and the majority of AEs were tolerable. Fatal side effects were rare even with long-term treatment. Therefore, long-term MMI treatment with careful monitoring of drug-related AEs is a useful treatment option before definite treatment in children and adolescents with GD. Compliance with long-term medical therapy is also important for these subjects to achieve remission. Further research is needed to determine the safety and optimal duration of long-term ATD treatment in children and adolescents with GD.

## Data Availability Statement

The raw data supporting the conclusions of this article will be made available by the authors, without undue reservation.

## Ethics Statement

This study was approved by the Institutional Review Board of Samsung Medical Center (SMC 2021-01-051). Written informed consent from the participants’ legal guardian/next of kin was not required to participate in this study in accordance with the national legislation and the institutional requirements.

## Author Contributions

AS oversaw data collection, interpretation, management, and statistical analysis for this study. SK contributed to data analysis and interpretation, management, and drafting of the article. M-SK and JK developed the structure and arguments for the paper and were responsible for clinical data collection. IK, GB, and ES analyzed and interpreted the data. YC and JC measured, analyzed, and interpreted the data. SC contributed to the research design, data analysis and interpretation, the critical review of the manuscript and approval of the submitted paper. D-KJ also approved the submitted paper. SC and D-KJ contributed equally to this work. All authors contributed to the article and approved the submitted version.

## Conflict of Interest

The authors declare that the research was conducted in the absence of any commercial or financial relationships that could be construed as a potential conflict of interest.
