# Supplementary material for: Long-Term Antithyroid Drug Treatment of Graves’ Disease in Children and Adolescents: A 20-Year Single-Center Experience
Source: Front Endocrinol (Lausanne). 2021 Jun 14;12:687834. doi: 10.3389/fendo.2021.687834 (PMC8236938; doi:10.3389/fendo.2021.687834)
Supplement: Supplementary file 1 [file DataSheet_1.docx]

Supplementary Material

# Standardization methods of thyroid function tests (TFT) considering the reference ranges of two different analysis kits.

# TFT was measured by two methods: radioimmunoassay using an RIA kit (Immunotech Inc., Praha, Czech Republic) and chemiluminescence immunoassay using ADVIA CENTAUR XP (Seimens Healthcare Diagnostics, Massachusetts, USA). The reference ranges of TFT using radioimmunoassay are fT4 0.64–1.72 ng/dL (female), 0.79–1.86 ng/dL (male), TSH 0.30–6.50 μIU/mL (female), 0.30–6.00 μIU/mL (male), and Total T3 76–190 ng/dL. The reference ranges using the chemiluminescence immunoassay are fT4 0.89–1.80 ng/dL, TSH 0.64–6.27 μIU/mL, and Total T3 60–181 ng/dL. Although different reference ranges were obtained with each assay kit, these differences were insignificant. For accurate statistical analysis, the TFT values measured in each analysis kit were standardized by the following formula considering the appropriate reference ranges of each. The values of the radioimmunoassay were converted to the chemiluminescence immunoassay values using the following formula. The reference range of the chemiluminescence immunoassay was used as the main reference range.

$${Value}_{St}=\left[ \frac{({Value}_{CIA}-{Min}_{CIA})}{({Max}_{CIA}-{Min}_{CIA})}\times({Max}_{RIA}-{Min}_{RIA}) \right]+{Min}_{RIA}$$

where $\mathrm{Value}_{\mathrm{St}}$= standardized value, $\mathrm{Value}_{\mathrm{CIA}}$= value of the chemiluminescence immunoassay (CIA), $\mathrm{Min}_{\mathrm{CIA}}$= minimum of the reference range of the CIA, $\mathrm{Max}_{\mathrm{CIA}}$= maximum of the reference range of the CIA, $\mathrm{Max}_{\mathrm{RIA}}$= maximum of the reference range of the radioimmunoassay (RIA), and $\mathrm{Min}_{\mathrm{RIA}}$= minimum of the reference range of the RIA.

ATA and AMA were measured with anti-Tgn (Brahms, Berlin, Germany). The reference ranges of both ATA and AMA are 0-100 U/mL before February of 2010 and 0-60 U/mL after February of 2010. Two TRAb-assays were used during the study period. During the first part of the study (before March of 2011), we used a TRAK-Assay (Brahms, Berlin, Germany; reference range, < 10% negative, 10-15% borderline, > 15% positive), which is a first-generation assay and a conventional radioreceptor assay using porcine thyrocytes membrane. Thereafter (after March of 2011), we used a TRAK human RIA (Brahms; reference range, < 1.0 IU/L negative, 1.0-1.5 IU/L borderline, > 1.5 IU/L positive), which is a second-generation assay and a coated tube radioimmunoassay using recombinant human TSH receptor.

# Supplementary Tables

## Table 1. Comparison of clinical and biochemical characteristics between the follow-up group and the lost to follow-up group

| **Parameter** | **Total subjects**  **(*n* = 187)** | **Follow-up**  **(*n* = 134)** | **Lost to**  **follow-up**  **(*n* = 53)** | ***P*-value*** |
| --- | --- | --- | --- | --- |
| Sex  Male, *n* (%)  Female, *n* (%) | 32 (17.1%)  155 (82.9%) | 22 (16.4%)  112 (83.6%) | 10 (18.9%)  43 (81.1%) | .688^=^ |
| Past medical history of AID (%)  Positive, *n* (%)  Negative, *n* (%) | 5 (2.7%)  182 (97.3%) | 4 (3.0%)  130 (97.0%) | 1 (1.9%)  52 (98.1%) | >.999^‡^ |
| Family history of AITD (%)  Positive, *n* (%)  Negative, *n* (%) | 65 (34.8%)  122 (65.2%) | 46 (34.3%)  88 (65.7%) | 19 (35.8%)  34 (64.2%) | .844^=^ |
| Graves’ ophthalmopathy (%)  Yes, *n* (%)  No, *n* (%) | 60 (32.1%)  127 (67.9%) | 43 (32.1%)  91 (67.9%) | 17 (32.1%)  36 (67.9%) | .999^=^ |
| Goiter (%)  Yes, *n* (%)  No, *n* (%) | 145 (77.5%)  42 (22.5%) | 101 (75.4%)  33 (24.6%) | 44 (83.0%)  9 (17.0%) | .259^=^ |
| Age at diagnosis (years) | 12.9 ± 3.2 | 12.9 ± 3.3 | 12.9 ± 2.9 | .910^+^ |
| BMI score at diagnosis | 18.9 ± 3.4 | 18.6 ± 3.5 | 19.6 ± 3.1 | .058^+^ |
| Duration of ATD treatment (years) | 4.7 ± 3.4 | 4.8 ± 3.6 | 4.3 ± 2.7 | .539^+^ |
| Total T3 at diagnosis (pg/mL)  (*n* = 147) | 468.6 ± 214.0 | 466.3 ± 213.9 | 475.6 ± 216.9 | .780^+^ |
| FT4 at diagnosis (ng/dL)  (*n* = 137) | 4.6 ± 1.9 | 4.6 ± 1.8 | 4.7 ± 2.0 | .989^+^ |
| ATA at diagnosis (U/mL)  (*n* = 146) | 631.4 ± 2131.1 | 559.1 ± 2101.7 | 836.7 ± 2228.2 | .377^+^ |
| AMA at diagnosis (U/mL)  (*n* = 149) | 2158.7 ± 3958.8 | 1839.6 ± 2807.2 | 3058.8 ± 6108.0 | .310^+^ |

*Data are expressed as mean ± SD.*

*^*^ Significant association was classified as P < 0.05.*

*^=^Chi-square test; ^‡^Fisher’s exact test; ^+^Mann–Whitney U test*

*Abbreviations: AID, autoimmune disease; AITD, autoimmune thyroid disease; BMI, body mass index; ATD, Antithyroid drugs; T3, triiodothyronine; FT4, free thyroxine; ATA, antithyroglobulin antibody; AMA, antimicrosomal antibody; SD, standard deviation*

## Table 2. Comparison of TRAb between the remission group and the non-remission group

| **Parameter** | **Remission**  **(*n* = 53)** | **Non-Remission**  **(*n* = 96)** | **HR (95% CI)** | ***P*-value** |
| --- | --- | --- | --- | --- |
| TRAb at diagnosis (%)^‡^  (*n* = 68) | 66.0 ± 20.6 (32) | 67.1 ± 21.7 (36) | 1.018 (0.542 – 1.914) | 0.328^+^ |
| TRAb at diagnosis (U/L)^‡^  (*n* = 81) | 20.8 ± 27.8 (21) | 36.6 ± 46.5 (60) | 1.054 (0.629 – 1.765) | 0.101^+^ |

*Data are expressed as mean ± SD.*

*^‡^Two TRAb-assays were used during the study period. During the first part of the study (before March of 2011), the TRAK-Assay (reference range, < 10% negative, 10-15% borderline, > 15% positive) was used. Thereafter (after March of 2011), TRAK human RIA (reference range, < 1.0 IU/L negative, 1.0-1.5 IU/L borderline positive, > 1.5 IU/L positive) was used.*

*^+^* *Cox regression model was used.*

*Abbreviations: HR, hazard ratio; CI, confidence interval; TRAb, thyrotropin receptor antibody; SD, standard deviation*
